# Enteric Fever Unmasking Hemoglobin SC Disease

**DOI:** 10.4269/ajtmh.21-0349

**Published:** 2021-06-21

**Authors:** Catalina Arango-Ferreira, Hardenson Rodríguez-González, Leidy J. Londoño-Restrepo

**Affiliations:** 1 Division of Pediatric Infectious Diseases, Hospital San Vicente Fundación, Medellín, Colombia;; 2 Department of Pediatrics, University of Antioquia, Medellín, Colombia;; 3 Department of Pediatrics, University of Antioquia, Medellín, Colombia

A 7-year-old Black girl from rural Colombia was admitted with 5 days of right elbow pain, fever, asthenia, hyporexia, and coluria. She presented with contusive trauma of the aforementioned elbow 2 months before admission. She had tachycardia (144/minute), fever (38°C), tachypnea (75/minute), icterus, respiratory distress, hepatosplenomegaly, and diffuse abdominal pain. Right elbow erythema, edema, and pain were evident. The initial complete blood count test revealed thrombocytopenia (76,000/uL), leukocytes (5,100/uL), anemia (hemoglobin [Hb], 4.3 g/dl), cholestasis, and discrete elevation of the aminotransferase levels (total bilirubin, 14.63 mg/dl; direct bilirubin, 10.3 mg/dl; aspartate transaminase, 175 U/liter; alanine aminotransferase, 128 U/liter). Because of symptomatic anemia, she received a red blood cell transfusion.

**Figure 1.**
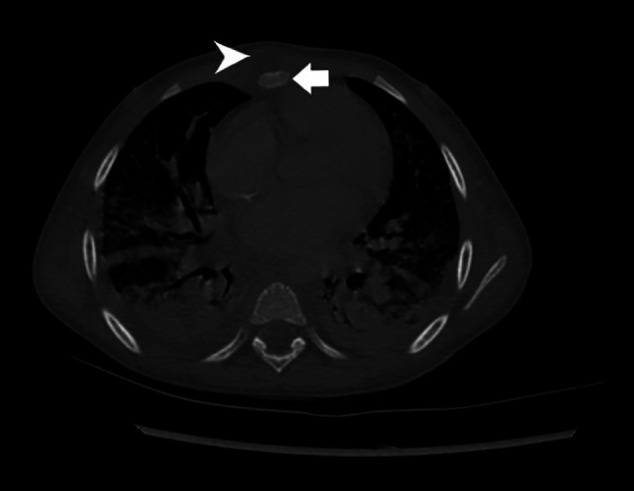
Chest computed tomography. White arrow indicates osteomyelitis of the body of the sternum. White arrowhead indicates thoracic abscess anterior to the sternum. Consolidation caused by multifocal pneumonia is also evident.

**Figure 2.**
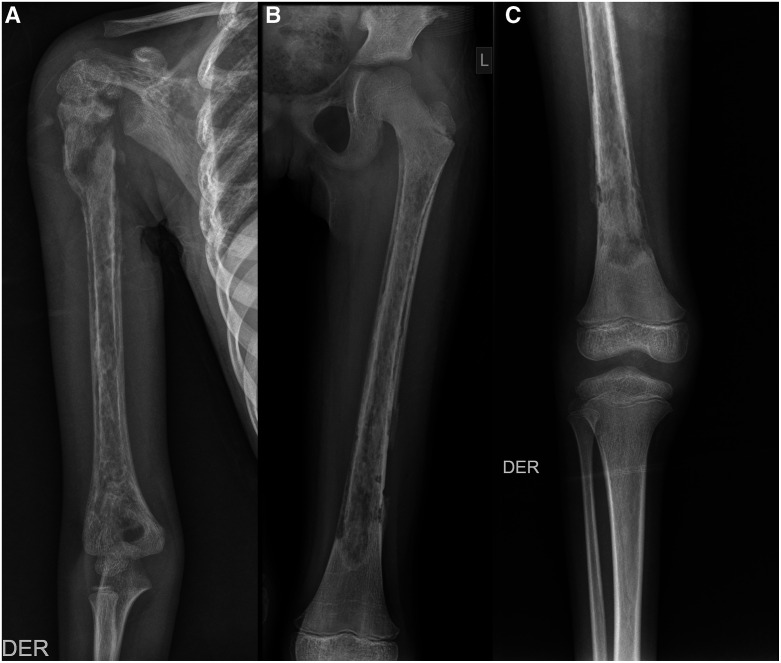
(**A**) X-ray examination of the right humerus shows osteomyelitis with a pathological fracture of the metaphysis of the humerus and glenohumeral subluxation. (**B**) X-ray examination of the left femur shows osteomyelitis. (**C**) X-ray examination of the right femur shows osteomyelitis.

**Figure 3.**
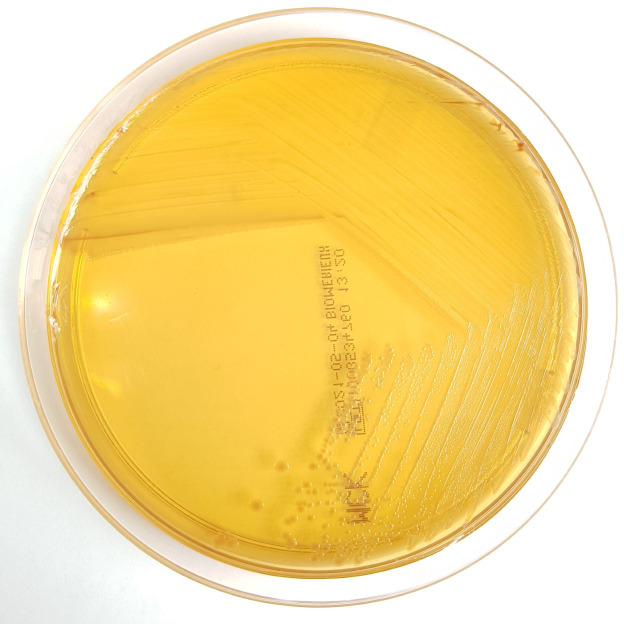
Petri dish with *Salmonella* spp. culture. This figure appears in color at www.ajtmh.org.

Her mother reported a history of jaundice and anemia during pregnancy, without a specific diagnosis. High-flow oxygen, fluid resuscitation, empiric broad-spectrum antibiotics (vancomycin and ceftriaxone), and vasopressors were initiated. The anemic syndrome was progressive and required multiple red blood cell transfusions. Viral hepatitis (A, B, C) and hemoparasites were ruled out. She developed pneumonia, a parasternal abscess, sternal osteomyelitis (Figure 1), recurrent fevers with multiple sites of osteomyelitis (right humerus, bilateral femurs, and iliac bones), left thigh myositis, and hip arthritis (Figure 2) that required surgical drainage. Matrix-assisted laser desorption ionization time of flight identified *Salmonella* sp. in blood, bone, and soft tissue cultures. Vitek2^®^ further identified wild-type *Salmonella enterica* spp. *enterica* (Figure 3). The antibiotic spectrum was narrowed to ciprofloxacin. Because of this severe disease course, along with the isolated microorganism, progressive hemolytic anemia, and family history, falciform anemia was suspected. Hb SC disease (SC) with positive sickling test and post-transfusion hemoglobin electrophoresis (HbA, 81.9%; HbS, 8.2%; HbA2, 3.4%; HbC, 6.5%) was confirmed. She was discharged and advised to attend follow-up appointments.

Enteric fever is a severe febrile illness caused by Gram-negative bacilli *Salmonella enterica*,* subspecies Typhi*, and *Paratyphi A*,* B*, and* C.* It is endemic in regions with poor sanitary and hygienic conditions. In 2017, 14.3 million cases were reported worldwide; 55.9% occurred in individuals younger than 15 years.^1^ It includes multiorgan manifestations beyond gastrointestinal symptoms, such as headache, myalgia, dry cough, and jaundice.^2^ Differential diagnoses include oncologic (leukemia), rheumatologic (juvenile idiopathic arthritis), and infectious diseases that present with cytopenia, organomegaly, and prolonged fevers.^3^ Sickle cell anemia predisposes to a severe course of the disease, with usually milder forms in SC hemoglobinopathy. Multisystemic pain, including that affecting the intestinal capillaries, predisposes to low-grade bacteremia. Bone infarcts serve as the perfect niche for bacterial seeding, with the possibility of systemic dissemination during secondary bacteremia.^4^ *Salmonella* spp. as the cause of sternal osteomyelitis is infrequent, and this is the third reported case.^5^
